# Isolation and Characterization of a Type 2 Vaccine-Derived Poliovirus from Environmental Surveillance in China, 2012

**DOI:** 10.1371/journal.pone.0083975

**Published:** 2013-12-26

**Authors:** Zexin Tao, Yong Zhang, Yao Liu, Aiqiang Xu, Xiaojuan Lin, Hiromu Yoshida, Ping Xiong, Shuangli Zhu, Suting Wang, Dongmei Yan, Lizhi Song, Haiyan Wang, Ning Cui, Wenbo Xu

**Affiliations:** 1 Academy of Preventive Medicine, Shandong University, Jinan, People's Republic of China; 2 Shandong Provincial Key Laboratory of Infectious Disease Control and Prevention, Shandong Center for Disease Control and Prevention, Jinan, People's Republic of China; 3 WHO WPRO Regional Polio Reference Laboratory and State Key Laboratory for Molecular Virology and Genetic Engineering, Institute for Viral Disease Control and Prevention, Chinese Center for Disease Control and Prevention, Beijing, People's Republic of China; 4 School of Public Health, Shandong University, Jinan, People's Republic of China; 5 Department of Virology II, National Institute of Infectious Diseases, Tokyo, Japan; 6 Department of Preventive Medicine, College of Basic Medical Sciences, Shandong University of Traditional Chinese Medicine, Jinan, People's Republic of China; The Scripps Research Institute, United States of America

## Abstract

Environmental surveillance of poliovirus on sewage has been conducted in Shandong Province, China since 2008. A type 2 vaccine-derived poliovirus (VDPV) with 7 mutations in VP1 coding region was isolated from the sewage collected in the city of Jinan in December 2012. The complete genome sequencing analysis of this isolate revealed 25 nucleotide substitutions, 7 of which resulted in amino acid alteration. No evidence of recombination with other poliovirus serotypes was observed. The virus did not lose temperature sensitive phenotype at 40°C. An estimation based on the evolution rate of the P1 coding region suggested that evolution time of this strain might be 160–176 days. VP1 sequence analysis revealed that this VDPV strain is of no close relationship with other local type 2 polioviruses (n = 66) from sewage collected between May 2012 and June 2013, suggesting the lack of its circulation in the local population. The person who excreted the virus was not known and no closely related virus was isolated in local population via acute flaccid paralysis surveillance. By far this is the first report of VDPV isolated from sewage in China, and these results underscore the value of environmental surveillance in the polio surveillance system even in countries with high rates of OPV coverage.

## Introduction

Polioviruses (PVs) have three serotypes and belong to the *Enterovirus* genus, *Picornaviridae* family. Their infection is known to be associated with acute paralytic poliomyelitis. The global incidence of poliomyelitis has dropped by more than 99 per cent since the Global Polio Eradication Initiative (GPEI) was launched in 1988 [1]. No case due to type 2 wild poliovirus (WPV) has been identified since 1999 and the remaining two serotypes are limited to just a small number of endemic regions [2], [3].

However, the GPEI has faced obstacles to the desired target. One is the re-emergence of WPV in previously polio-free countries, such as the WPV1 importation to mainland China in 2011. Another is the emergence of vaccine-derived polioviruses (VDPVs) (>1% divergent [PV1 and PV3] or >0.6% divergent [PV2]). Since the live, attenuated oral poliovirus vaccine (OPV) is a main tool used in polio eradication efforts, rare paralytic poliomyelitis cases caused by VDPVs can occur when neurovirulence reverses as the result of mutations. VDPVs have the potential for sustained circulation in areas with low OPV coverage and many outbreaks of circulating VDPVs (cVDPVs) were reported worldwide in recent years [4], [5]. In Mainland China, VDPVs have also been identified through the acute flaccid paralysis (AFP) surveillance system [6]. One of the interesting observations from the recent outbreaks is that most of the cVDPVs reported in these outbreaks is Sabin2 associated. Most of the type2 VDPVs isolated from the field contain the G481A and U2909C mutation encoding a Ile to Thr substitution in VP1. These two mutations appear to be responsible for the neurovirulence reversion of the Sabin2 VDPVs [7].

The World Health Organization (WHO) strategy for monitoring the wild type and mutated vaccine poliovirus is to identify virus isolates from AFP cases and their contacts. Environmental surveillance serves as a supplementary method to monitor the PV transmission in human populations by examining sewage specimens which have been potentially contaminated by human feces [8]. Surveillance for enteroviruses in sewage samples has been conducted in our laboratory since 2008. For the first time, a type 2 VDPV strain E12–221 was isolated from the sewage collected in December, 2012. In this report, we describe the genomic characterization, temperature sensitivity phenotype and phylogenetic analysis of this virus, and discuss the significance of environmental surveillance in GPEI.

## Materials and Methods

### Ethics Statement

The ethical approval was given by Ethics Review Committee of the Shandong Center for Disease Control and Prevention, and the study was conducted in compliance with the principles of the Declaration of Helsinki. Written informed consents for the use of their clinical samples were obtained from the parents or legal guardians of the patients. The permission for each sampling location was issued by Shandong Provincial Environmental Protection Department.

### Shandong Province and sampling sites

Shandong is a coastal province located in the eastern part of China with an area of 156,700 km^2^ and a population of 95.79 million (2010 census data). Jinan is the capital city of Shandong Province. Its metropolitan area and population is 296 km^2^ and 2.6 million, respectively. Linyi is a city with frequently documented large outbreaks of enteroviral diseases in recent years [9]–[11]. Its metropolitan area and population is 178 km^2^ and 1.9 million, respectively. The inlets to the sewage treatment plants of the two cities, namely, Jinan Everbright Water and Linyi Shouchuang Water, were chosen as the sampling sites.

### Sampling and concentration

Collected monthly in Jinan and semimonthly in Linyi, the samples were from the inlet collector canal by grab sampling method between 2–3 pm. Approximately 1 liter of sewage sample was collected from flowing sewage by a stainless plastic bucket and maintained at about 4°C during sample transport, storage (<24 h), and processing.

Sewage samples were concentrated by membrane absorption/elution method as described previously [12], [13]. Briefly, the sewage samples were centrifuged at 3000×*g* for 30 min at 4°C. 2.5 M MgCl_2_ was added to the supernatant to a final concentration of 0.05 M. The pH value was adjusted to 3.5 by 0.5 M hydrochloric acid. Then the solution was filtered through a 0.45-µm-pore-size, mixed cellulose ester membrane filter (Advantec, Tokyo, Japan) by positive pressure pump. Absorbents on the filter were then eluted with 10 ml 3% beef extract solution followed by ultrasonication for 5 minutes, and the solution was centrifuged at 4000×*g* for 30 min. Subsequently the supernatant was filtered through a 0.22-µm-pore-size filter and was ready for cell inoculation.

### Virus isolation and serotyping

L20B, RD and HEp-2 cell lines were used for virus isolation. All these cell lines were gifts from the WHO Global Specialized Laboratory in USA and were all originally purchased from the American Type Culture Collection (ATCC). A total of 200 µl of concentrated solution was added to each of the cell culture tube (18 tubes of each cell line for one sewage sample). Subsequently, the tubes were kept in 36°C incubator for 7 days and were examined daily. After 7 d, the tubes were frozen and thawed and re-inoculated into L20B, RD and HEp-2 cell lines. If a complete cytopathic effect (CPE) was observed in RD or HEp-2 cell lines, the cells in the tube were frozen and thawed and inoculated into L20B cells for isolation of PV.

According to standard protocols recommended by the WHO [14], PV serotyping was carried out via micro-neutralization assays in 96-well tissue culture plates using polyclonal antisera against PV types 1, 2 and 3 (National Institute for Public Health and the Environment, RIVM, the Netherlands). The antisera-virus mixtures were incubated for 1 h at 36°C. Subsequently, suspensions of cells were added to the plate which was subsequently examined daily for the presence of CPE. The antiserum that prevented the development of CPE indicated the identity of the virus.

### PV isolates from AFP surveillance

The specimens from AFP cases in Shandong Province were collected between 2012 and June 2013 and processed according to standard protocols recommended by the WHO [14].

### VP1 sequencing and phylogenetic analysis

Total RNA was extracted from 140 µl of the infected cell culture using QIAamp viral RNA mini kit (Qiagen, Valencia, CA, USA) according to the manufacturer's recommended procedure. RT-PCR was performed using Access RT-PCR System (Promega, USA). Primer pair UG1/UC11 [15] was used to amplify the entire VP1 coding region. PCR products were purified and sequenced directly with the BigDye Terminator v3.0 Cycle Sequencing kit (Applied Biosystems, Foster City, CA) by ABI 3130 genetic analyzer (Applied Biosystems). The PCR products were sequenced in both directions to avoid possible ambiguous nucleotides.

Nucleotide sequence alignments were carried out by BioEdit 7.0.5.3 software [16]. Phylogenetic trees were constructed by Mega 4.0 [17] using neighbor-joining method after estimation of genetic distance using the Kimura two-parameter method [18]. A bootstrapping test was performed with 1,000 duplicates, and the transition/transversion rate was set at 2.0.

### Full-length genome amplification

The complete genome of VDPV2 strain E12–221 was amplified by two long-distant PCR reactions using the TaqPlus Precision PCR system (Stratagene, USA). The primer pairs Y7/7500A and 0010S48/Q8 [19], [20] were used to amplify a 5.28 kb and 3.57 kb fragment, respectively. The combined sequences of the two fragments yielded the entire genome sequence. Sequencing were performed with primers described previously [6].

### Estimation of the date of the initial OPV administration

The time interval between the date of OPV administration and the date of sampling of the VDPV2 strain E12–221 was estimated via the calculation of *Ks* (synonymous substitutions per synonymous site) and *Kt* (all the substitutions per site) values in the P1 coding region. It is assumed that the evolution rates are 0.032 synonymous substitutions per synonymous site per year and 0.011 total substitutions per site per year [21].

### Temperature sensitivity

Temperature sensitivity of strain E12–221 was tested on monolayered RD cells in 6-well plates as described before [22]. 200 µl of virus stocks was inoculated onto the cells. After absorption at 36 or 40°C for 1 h, the supernatant was removed, and 2.5 ml of maintenance medium was added and incubated at 36 or 40°C, separately. After 8, 24, and 48 h, the plates were harvested, and the CCID_50_ were calculated in 96-well plates. More than 2 logarithms reduction of the titers at different temperatures was considered to be temperature sensitive. In order to minimize experimental error, the assay was repeated three times.

### Reported vaccination rate and AFP surveillance data

The vaccination rate of Jinan city in 2012 was obtained from the National Immunization Information Management System. The AFP surveillance data was obtained from the China Information System for Diseases Control and Prevention.

### Rapid assessment of vaccination rate

Rapid assessment was conducted in the 10 counties of Jinan city from January 21 to 24, 2013. Two towns were randomly selected for each county, three villages were randomly selected for each town, and six children <4 years of age in each village were investigated for the OPV immunization history.

### Nucleotide sequence accession numbers

The complete genome sequence of VDPV2 isolate E12–221 and VP1 sequences of other PV2 strains from sewage described in this study were deposited in the GenBank database under the accession numbers KF656732 and KF666568–KF666633, respectively.

## Results

### Analysis of the genome sequence

A virus strain named E12–221 was isolated from the sewage collected in Jinan in December 2012. Neutralization test showed that it was a type 2 poliovirus. Based on the full-length genome sequencing analysis, we found that, compared with Sabin 2 strain, E12–221 contains 25 nucleotide substitutions with 7 mutations resulting in amino acid alteration (Table 1). It should be noted that, in strain E12–221, the nucleotide A at position 481 in 5′ noncoding region has been mutated to G, which is a neurovirulent reversion mutation usually observed in the VDPV-associated paralytic poliomyelitis cases [7]. In the VP1 coding region of E12–221, there are 7 substitutions, 4 of which lead to amino acid change. However, the Ile at position of 143 in VP1, the other neurovirulent determinant of Sabin 2, didn't mutated. In addition, no recombination event was observed.

**Table 1:** Nucleotide and amino acid substitutions in the sewage isolate E12–221 in comparison to Sabin 2.

Region	Nucleotide			Amino acid		
	Position*	Sabin 2	E12–221	Position*	Sabin 2	E12–221
5′ NTR	398	U	C			
	**481**	**A**	**G**			
	735	C	U			
VP4	786	C	U			
VP2	960	A	G			
	993	A	G			
VP3	1905	G	A			
	2005	A	G	420	T	A
	2026	U	C			
	2256	A	G			
VP1	2548	U	A	601	S	T
	2557	A	G	604	S	G
	2595	C	U			
	2986	A	G	747	K	E
	3120	A	U			
	3145	U	C	800	S	P
	3339	U	C			
2C	4158	U	C			
	4188	C	U			
	5031	U	C			
3C	5578	G	A	1611	A	T
	5946	C	U			
3D	6209	A	G	1821	K	R
	6390	U	A			
	6765	C	U			

Nucleotide and amino acid positions are numbered according to Sabin 2 (AY184220).

The nucleotide position described as being involved in Sabin 2 attenuation is in bold.

### Estimation of the evolution time of strain E12–221

The approximate evolution time of the VDPV2 strain after the initial vaccine dose was estimated by its difference from Sabin 2 strain in the P1/capsid coding region. The *Ks* and *Kt* values for P1 coding region was 1.4% and 0.53% respectively. Compared with the reported evolution rate of 3.2% (*Ks*) and 1.1% (*Kt*) in P1 region per year [21], we estimated the evolution time of the virus was 160 days (from the *Ks* estimate) or 176 days (from the *Kt* estimate). Assuming a one-week efflux of the virus in the sewage network, the administration time of the parental OPV might be June 2012. Nevertheless, it had to be admitted that the evolution time was just an estimate. The actual origin of the virus and its evolutionary experience in the sewage network might differ from the deduction.

### Temperature sensitivity

The temperature sensitivity assay of VDPV2 strain E12–221 showed that there is a titer reduction of more than 2 logarithms at different temperatures (Table 2), suggesting that, similar to Sabin 2, VDPV2 strain E12–221 still maintains the temperature sensitive phenotype. The three times of repeat showed the similar results.

**Table 2:** Temperature sensitivity of VDPV2 strain E12–221 and Sabin 2 strain.

Virus	Titers at 37°C			Titers at 40°C			Log titer reduction		
	8 h p.i.	24 h p.i.	48 h p.i.	8 h p.i	24 h p.i.	48 h p.i.	8 h p.i	24 h p.i.	48 h p.i.
Sabin 2	8.0	8.0	7.9	5.5	5.0	5.5	2.5	3.0	2.4
E12–221	7.9	8.5	8.0	5.9	5.6	5.0	2.0	2.9	3.0

The titers and its reduction at different temperatures are presented as logarithm 10.

### Phylogenetic analysis

**Figure 1:**
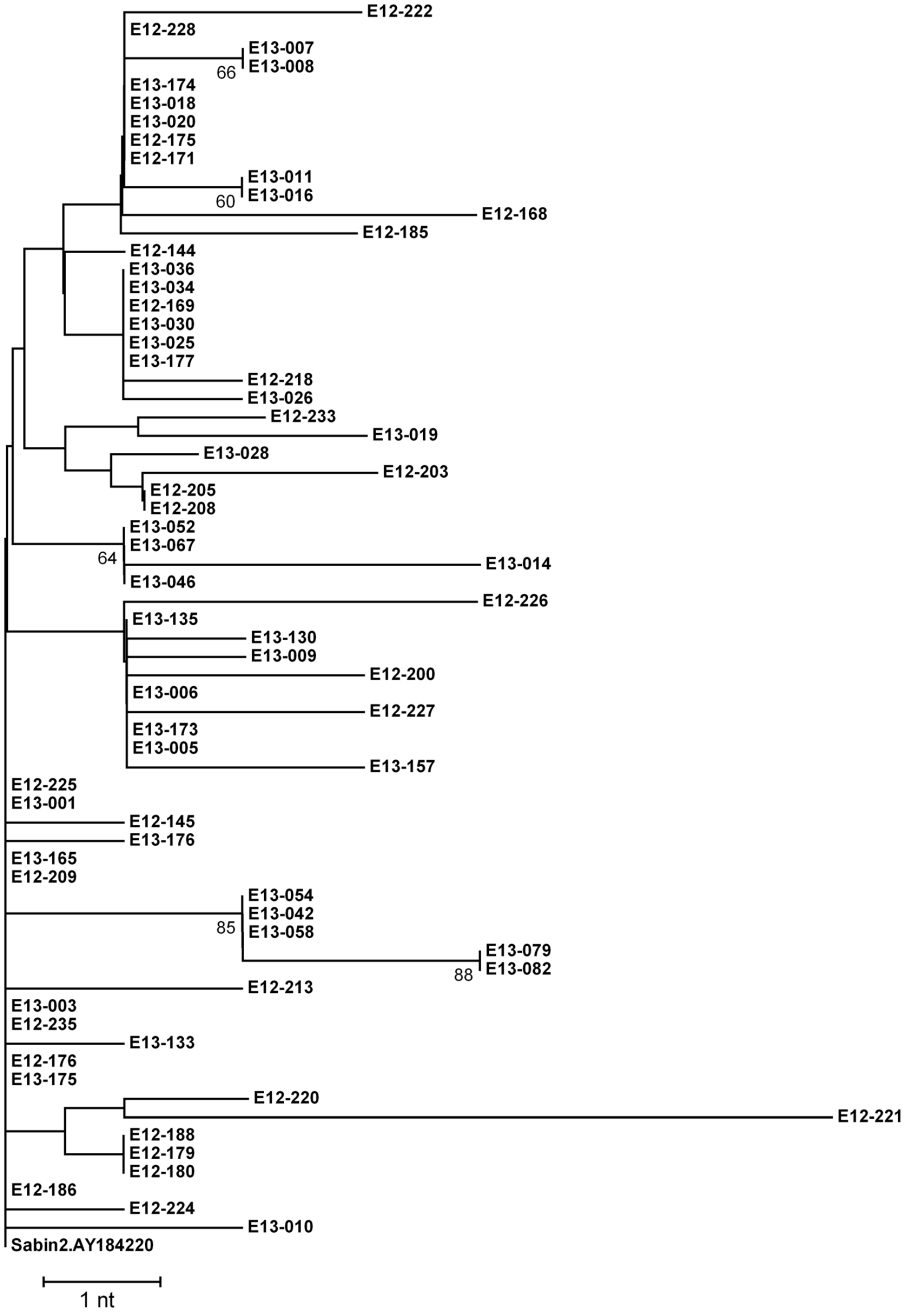
Phylogenetic tree based on VP1 sequences of all environmental polioviruses. The polioviruses in the tree were isolated from sewage in Shandong Province from May 2012 to June 2013. Strain E12–221 is a VDPV2 strain isolated in December 2012. The isolates are represented as E(SampleYear)-(serial no). The percentage number at the node corresponds to the resampling value, and the values lower than 60% are not shown.

According to the estimated evolution time of 160 or 176 days of VDPV2 strain E12–221, the VP1 coding regions of all other PV2 strains isolated from sewage collected from May to December 2012 (n = 28) were sequenced. Also, the VP1 coding regions of the environmental PV2 strains isolated from sewage collected from January to June 2013 (n = 38) were sequenced. The results showed they were all Sabin strains. The VP1 substitution numbers of these strains only ranged from 0 to 4 and no other VDPV strain was identified. The phylogenetic tree based on the VP1 sequences of these environmental strains (n = 67, including strain E12–221) revealed that no close relationship was observed between strain E12–221 and any other strains isolated six months before and after (Figure 1). Only four environmental strains shared identical substitutions with this VDPV2 strain. Strains E12–179, E12–180 and E12–188 isolated in October 2012 shared one substitution (A to G at position 2557 of the genome) with the strain E12–221. Strain E12–220 isolated in December 2012 shared one substitution (A to G at position 2986) with the strain E12–221.

**Figure 2:**
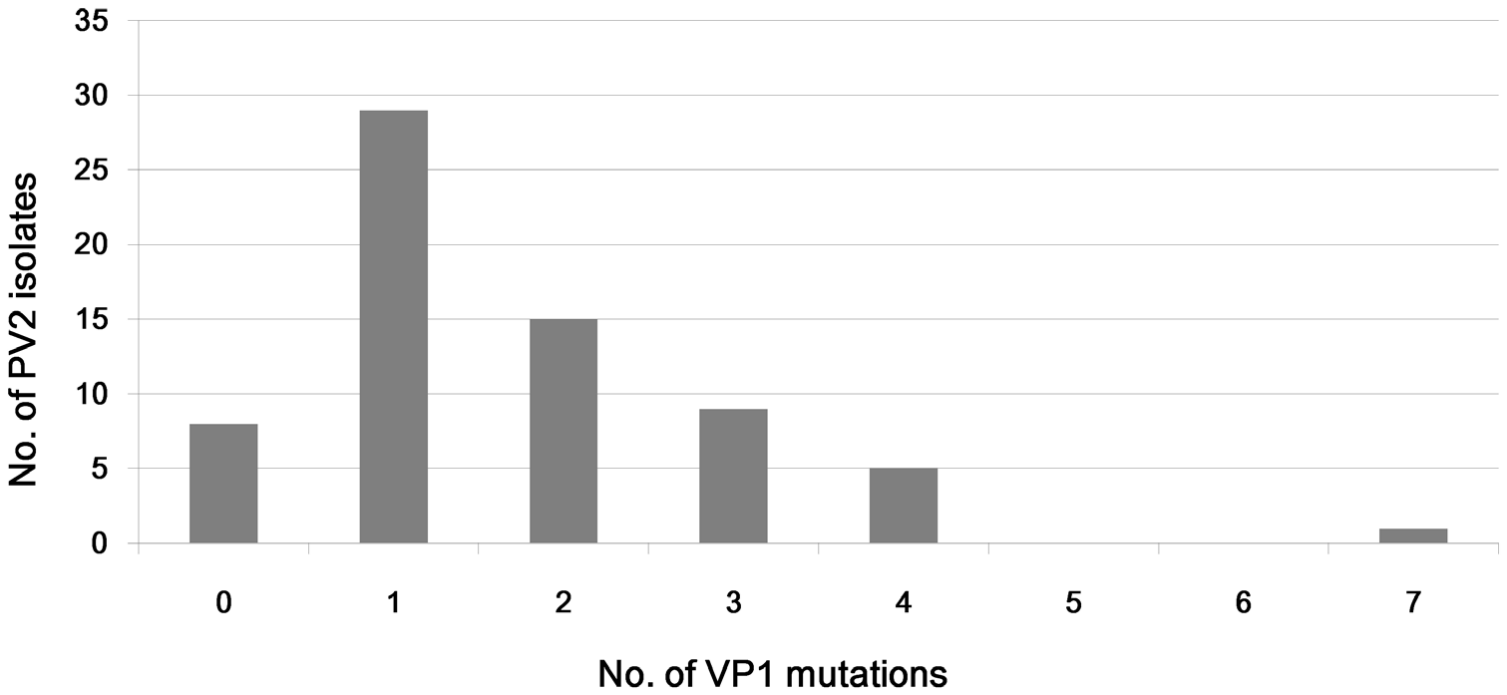
VP1 mutation numbers of environmental PV2 in Shandong from May 2012 to June 2013.

The mutation numbers in VP1 nucleotide and amino acid sequences of all environmental PV2 strains in this study was illustrated in Figure 2. PV2 strains with 1 nucleotide or amino acid mutation were the most frequently isolated. A total of 29 amino acid substitutions were found in the 67 environmental strains in VP1 sequence, most of which located near the amino terminal of VP1. Six hot sites for amino acid substitution were identified (Table 3). Unsurprisingly, the Ile143Thr mutation was one of these hot sites.

**Table 3:** Hot sites of amino acid substitutions in the VP1 of 67 PV2 isolates in comparison to Sabin strain.

VP1 amino acid position	Substitution	Number of isolates
26	S to G	4
36	A to T	5
40	E to D	4
43	A to V	10
	A to T	1
56	V to E	5
143	I to T	13
	I to N	9
	I to V	2
	I to S	1

### Reported vaccination rate and AFP surveillance data

The vaccination rate of OPV dose 1, 2, 3, and 4 in Jinan city in 2012 is reported to be 99.75%, 99.67%, 99.75% and 99.70%, respectively. The incidence of AFP cases in Jinan city is reported to be 5.05, 4.80 and 5.92 per 100 000 in 2010, 2011 and 2012, respectively.

### Rapid assessment of vaccination rate

A total of 1800 children in Jinan city were investigated, and 98.89% had completed the OPV immunization.

## Discussion

Since the last WPV associated paralytic poliomyelitis patient in 1991, Shandong Province had maintained polio free for 22 years. However, VPDV had been detected in Shandong AFP surveillance system in 2007 (P1, 9 nt; P1, 13 nt), 2009 (P2, 11 nt) and 2011 (P1, 10 nt), respectively. They were all ambiguous VPDV (aVDPV) which were clinical isolates from persons with no known immunodeficiency [4].

As a supplemental method to AFP surveillance for global poliomyelitis eradication, environmental surveillance is of great importance in investigating the circulation of WPV or VDPV [23]–[26]. Enterovirus environmental surveillance had been conducted in Shandong Provincial Poliovirus Laboratory since February 2008. Till June 2013, a total of 351 PVs has been isolated but no WPV was detected. Currently, OPV is still the major vaccine used for routine vaccination in Mainland China, therefore, resulting in the frequent detection of Sabin strains from sewage. By far strain E12–221 was the only VDPV strain detected in the environmental surveillance. In alignment with other environmental PVs collected six months before and after the collection date of the strain, no close relationship was found among themselves, suggesting the VDPV strain did not form a continuous transmission chain in local population. Also no closely related virus was obtained in local population via acute flaccid paralysis surveillance. So, the evidence available so far is still insufficient to conclude whether the VDPV was derived from importation from other regions or from local persons with primary immunodeficiency.

The A481G change in 5-noncoding region, along with an amino acid substitution (I143T) at nucleotide position 2909 (U to C) in VP1 have been well known to be responsible for the neuovirulent reversion and the loss of temperature sensitivity phenotype in type 2 VDPVs. In strain E12–221, only at position 481, nucleotide A has mutated to G but the I143T mutation at position 2909 (U to C) in VP1 was not observed even though a total of 7 amino acid mutations located in the VP3, VP1, 3C and 3D region were identified (Table 1). The lack of the I143G neurovirulent reversion mutation may help to explain why the E12–221 still maintains the temperature sensitive phenotype. Instead, for the 67 environmental strains, I143T in VP1 appears to be one of the hot spots of mutation (Table 3). It would be interesting to know, whether, among these strains with I143T, some of them also have the G481A mutation. Altogether, these results suggest that all these environmental type 2 vaccine strains have the tendency to evolve by gaining mutations. The acquirement of the two important ones at position 481 and 2909 is just a matter of time if their circulation in population cannot be interrupted by the high OPV coverage.

Shandong is a province with a high OPV coverage. According to the results of PV neutralization antibody examination in healthy population in 2011, the positive rate of PV1, 2, and 3 was 96.6%, 96.8% and 90.5%. The positive rate for children <2 years of age was 98.0%, 99.0% and 94.0%, respectively (data not shown). However, under such high OPV immunization circumstances, the VDPVs were still occasionally detected from AFP and environmental surveillance system in recent years, suggesting that the evolution of VDPV is not necessarily related to the poor level of local OPV immunization. The phylogenetic analysis revealed no close evolutionary relationship of other environmental PV2 to the VDPV in this study. Taken the relative less numbers of mutations of these VDPVs in Shandong (≤13 nt) into account, the high OPV coverage is suggested to play an important role in interrupting the further transmission of VDPV in local population. Hence, it is concluded that high OPV coverage cannot completely prevent the emergence of VDPV, but can block the occurrence of cVDPV.

Ever since the last WPV associated paralytic poliomyelitis cases was observed in Mainland China in 1994, four incidents of WPV importation has been reported. The most recent importation in Xinjiang had caused an outbreak that claimed 21 poliomyelitis cases. The poor local AFP surveillance network and OPV immunization may be the impulse to the spread of WPV. Environmental surveillance has been demonstrated to play an important role in early-warning of outbreak [27]. Previously, we reported the isolation of a type 3/type 2 recombinant poliovirus with chimeric capsid VP1 protein from sewage in Shandong in 2009 [28]. No such virus was identified from local AFP surveillance system at that time. In this study, the VDPV was isolated from sewage while no related AFP cases were reported. Hence, AFP surveillance combined with continuous environmental surveillance should be of great importance in improving the sensitivity of poliovirus detection.

## Conclusions

The present study describes the characterization a type 2 VDPV isolated from sewage in China. The surveillance of VDPVs will become increasingly important since OPV becomes the only remaining source of poliovirus infection in polio free regions. The results presented here confirmed the importance of environmental surveillance in GPEI.
